# Circulating RNA Transcriptome of Pregnant Women with TSH Just Above the Trimester-Specific Reference and its Correlation with the Hypertensive Phenotype

**DOI:** 10.1038/s41598-020-63040-5

**Published:** 2020-04-15

**Authors:** Andréa Harumy de Lima Hirata, Luiz Antônio de Jesus Rocha, Valdelena Alessandra da Silva, Robson José de Almeida, Lucas dos Santos Bacigalupo, Patrícia Varela, Leonardo Martins, João Bosco Pesquero, Humberto Dellê, Cleber Pinto Camacho

**Affiliations:** 1 Molecular Innovation and Biotechnology Laboratory, Postgraduate Program in Medicine, Universidade Nove de Julho (Uninove), São Paulo, SP Brazil; 2 Department of Obstetrics and Gynecology, Conjunto Hospitalar do Mandaqui, São Paulo, SP Brazil; 3 Center for Research and Molecular Diagnostic of Genetic Diseases, Department of Biophysics, Universidade Federal de São Paulo (UNIFESP), São Paulo, SP Brazil

**Keywords:** Predictive markers, Thyroid diseases

## Abstract

During gestation, a woman’s body undergoes physiological changes that alter thyroid function. Pregnant women with hypothyroidism may exhibit gestational complications, including hypertension and preeclampsia. We investigated differentially expressed genes (DEGs) in circulating RNAs from pregnant women with TSH levels just above the normal range to determine the impact of a mild elevation of TSH in pregnancy. We selected three women with healthy thyroid pregnancy (HTP), three pregnant women with gestational hypothyroidism (GHT), and three nonpregnant women (NPG) to construct transcriptome libraries. We also compared our results with data from the GEO dataset and DisGeNET. We identified 1500 DEG in GHT and 1656 DEG in HTP. From GEO dataset, we recognized 453 DEGs in trimester-specific plasma RNA, 1263 DEGs in placental tissues from healthy women, 1031 DEGs from preeclamptic uteroplacental tissues and 1657 DEGs from placental tissues from severely preeclamptic women. In this scenario, 12.26% and 12.86% genes were shared between these datasets in GHT and HTP, respectively. We stablished 62 genes in GHT DEGs related to hypertensive phenotype hallmarks. In conclusion, even in women with a mild TSH increment, we were able to detect some DEGs that could be associated with a hypertensive phenotype.

## Introduction

Circulating Ribonucleic Acid (RNA) represents a powerful strategy, less invasive and capable of real-time tracking diseases. The circulating protein codifying transcripts (mRNA, messenger RNA) may also provide clinically useful additional information when compared to the direct protein measurement^1^. This strategy is also sensible enough to detect fetal RNA circulating in the mother’s blood^2^. The use of corticotropin-releasing hormone (CRH) mRNA detection in pregnancy was also reported to be capable of working as a molecular marker for preeclampsia^3^. Nevertheless, the transcriptome analysis is efficient enough to offer much more information than the use of a single RNA. The target RNA detection strategies, like RNA-Seq, can be performed in a simple blood sample and can give us the quantitative expression of the coding and noncoding RNA from the mother, the placenta or the fetus in a unique and noninvasive way^4^.

During gestation, a woman’s body undergoes physiological changes that alter thyroid function and thyroid hormone metabolism. Most of these changes are related to the increased demand for thyroid hormone by the mother and the fetus, the placental degradation of the hormone and the increase in the thyroxin-binding globulin (TBG) concentration. TBG increases due to an estrogenic stimulus during pregnancy, and for this reason, free thyroxine (FT_4_) is highly influenced by the significant increase in the thyroxine (T_4_)-bound fraction. Additionally, an increase in human chorionic gonadotropin (hCG) stimulates thyroid function through homologous binding to the thyroid-stimulating hormone (TSH) receptor, promoting a physiological reduction of TSH levels. These physiological modifications are necessary to guaranty the maternal and fetal healthy^5^.

During a healthy pregnancy, an increase in the production of nitric oxide induced by estrogen and relaxin plays an essential role in vasodilation to supply the oxygen and nutrient demand of the fetus from the mother^6^. Taddei *et al*. studied subjects with subclinical hypothyroidism and demonstrated a reduction in nitric oxide release, leading to vasoconstriction from the impaired endothelium^7^. Therefore, altered concentrations of thyroid hormones in the body can alter the regulation of blood pressure^8^. Consequently, hypothyroidism was associated with an increase of 22% risk for gestational hypertension^9^. In a prospective study with 8012 pregnant women, 4.63% had hypothyroidism and the observed risk was 2.2 for gestational hypertension^10^. The secondary analysis of a prospective prenatal population-based study, evolving 26,197 women, also found an association of hypothyroidism with gestational hypertension and preeclampsia^11^. Another population-based cohort study with 571,785 women also showed an association between maternal hypothyroidism and gestational hypertension or severe preeclampsia, which seems to be related with a non‐consistent levothyroxine use^12^.

Nevertheless, there are some contradictory data, probably influenced by biological and methodological problems. Genetic and environmental factors such as inadequate iodine intake and vitamin D intake may cause variability regarding hypertension and preeclampsia outcomes in pregnant women with hypothyroidism^13–15^. The modifications on the definitions criterion of overt or subclinical hypothyroidism and gestational hypertension or preeclampsia may also influence the results^16–19^. Because of all those experimental variations and a considerable number of patients are necessary, studies with a low number of women included fail to prove this association.

Based on that, we decided to look at this problem with another perspective and use a circulating RNA approach. We built transcriptome libraries and called a differentially expressed genes (DEG), which may allow us to identify some physiopathological alteration indirectly through the circulating RNAs. Moreover, we compared our DEGs with data from healthy pregnant women and preeclampsia patients DEGs, and also with hypertensive disease databanks to identify common genes, which may uncover the mechanisms by which hypothyroidism leads to hypertension and to find biomarkers capable of predicting hypertension in pregnant patients with hypothyroidism. Additionally, we tried to find an association between our DEG from gestational hypothyroidism with maternal blood circulating fetal genes.

## Methodology

### Ethical approval and informed consent

The study followed the principles of the Declaration of Helsinki and the Institutional Review Board (IRB) of the University Nove de Julho. After approval of the IRB - numbers 665.331 (CAAE: 30746814.4.0000.5511) and 679.727 (CAAE: 30746814.4.3001.5551), all the participants provided written informed consent.

### Population

We selected three third trimester women with healthy thyroid pregnancy (HTP) and three third trimester pregnant women with gestational hypothyroidism (GHT) from a tertiary State Hospital. We also recruited three nonpregnant women (NPG) from a secondary health system outpatient clinic to construct transcriptome libraries. The women in the HTP group had to exhibit a TSH level under the trimester-specific reference range and could not have any disease. On the other hand, the GHT women must exhibit a stable TSH level above the trimester-specific reference range and could not have any other disease. At the beginning of this study, because of the necessary period to define thyroid function as “stable,” we decided to exclude pregnant women with TSH levels above the healthy adult reference range to avoid any damage to the mother or the fetus.

### Blood sampling, RNA extraction, and cDNA synthesis

Blood was collected for hormonal measurements and molecular analysis. Serum TSH was analyzed in a Cobas Roche Elecsys 600 instrument (Roche Diagnostics, IN, USA) with a detection sensitivity of 0.005 mIU/L and an interassay variance of 3.44%. The manufacturer’s reference range for TSH levels in pregnant women in the third trimester is 0.21 to 3.15 mIU/L. We also performed the assessment of serum TgAb (detection of 10–4000 mIU/L, reference range of less than 177.0 mIU/L), TPOAb (detection of 5–600 mIU/L, reference range of less than 123.0 mIU/L), and FT_4_ (detection of 0.3–100 pmol/L, reference range of 0.65–1.21 ng/dL) in an Elecsys 2010 Roche Diagnostics instrument (Roche Diagnostics).

A PAXgene blood RNA tube (QIAGEN, NL, DE) was used for the preservation of RNA molecules. The extraction of total RNA was performed with the PAXgene Blood RNA Kit (QIAGEN, NL, DE), and we followed the manufacturer’s protocol. Total RNA was quantified with a Qubit fluorometer 2.0 (Thermo Fisher Scientific, Waltham, Massachusetts, USA), and the Invitrogen SuperScript VILO cDNA Synthesis Kit (Thermo Fischer Scientific) was used for cDNA synthesis.

### Transcriptome library construction and genetic evaluation

The libraries were constructed with 22786 initial targets using the Ion AmpliSeq^TM^ Library Kit Plus (Thermo Fisher Scientific, Waltham, Massachusetts, USA) and target regions from the Transcriptome Human Gene Expression panel aligned against the genome reference hg19 version. The Ion PI™ Hi-Q™ OT2 Reagent 200 (Thermo Fisher Scientific) was used for emulsion PCR in a One Touch2™ Instrument (Thermo Fisher Scientific) Enrichment of template-positive ISPs was performed in an Ion OneTouch™ ES (Thermo Fisher Scientific with Dynabeads® MyOne™ Streptavidin C1 Beads (Ion Proton™ System) (Thermo Fisher Scientific), and subsequent sequencing was carried out with an Ion PI^TM^ Chip (Thermo Fisher Scientific) in an Ion Proton™ Sequencer (Thermo Fisher Scientific).

### Bioinformatics

The reads of each library were merged in a single count matrix. The matrix was used in the analysis with R Version 3.4.1, available at (https://www.R-project.or)^20^. The pcaExplorer package was used to access the quality control, and no library presented an aberrant behavior^21^. The multidimensional scale plot (MDS) was also used to guarantee the homogeneity of the libraries. We performed a three-group analysis between HTP, GHT and NPG. We used a fold change (logFC) cutoff of +1.0 and – 1.0 to select the relevant DEG from the FDR adjustment significant list of transcripts from the groups.

The normalization was performed with the Trimmed Mean of M-values (TMM) of the package edgeR^22^. The TMM excludes genes highly expressed and with a considerable variation, and the remaining are used for the normalization process^23^. Differences in expression were determined with edgeR v.3.16.5 considering differentially expressed genes as those genes with p-values <0.05 corrected via the FDR method^22^.

In the GEO dataset, limma 3.26.8 was used to construct the DEG list. We also excluded repeated genes or those whose gene symbols could not be identified. We considered significant genes with an adjusted p < 0.05.

### Biostatistics

The data are presented as medians with the minimum and maximum values. To compare the continuous variables among HTP, GHT and NPG, we used the Kruskal-Wallis test. A p < 0.05 was considered significant. The data were analyzed using IBM SPSS Statistics for Windows, Version 23.0, from IBM Corp., released in 2015 (Armonk, NY, USA). The Venn plot was constructed on the InteractiVenn website (interactivenn.net/)^24^.

### *In Silico* computational strategies to compare our DEG with other databanks

#### Trimester-specific plasma RNA fluctuation

To assess the variations in the plasma concentration of RNA for comparison with our DEG results, we used data from the GEO dataset (ncbi.nlm.nih.gov/geo) on the circulating RNA levels in the peripheral blood of eleven healthy women in the second and the same subjects in the third trimester (accession number: GSE56899). The clinical and laboratory characteristics of the subjects from GSE56899 were not available^25^. We sought to determine whether the observed variation was physiological, even in the GHT group, or if the fluctuation was in the opposite direction from the expected physiological process.

#### Trimester-specific placental RNA fluctuations

To assess the specific placental RNA variations during the studied period and for comparison with our DEG results to identify the circulating RNAs with a placental origin, we used data from four healthy placental tissues from the second trimester and four healthy placental tissues from term placentae (accession number: GSE9984). The subjects from GSE9984 did not have a history of chronical diseases or the use of medications. They also did not smoke, use drugs or abuse of alcohol^26^.

#### Placental preeclamptic tissue

To compare our DEGs with placental preeclamptic tissues, we used two sets of data. The first allowed us to compare the DEGs of a pool from decidual and a pool from placental tissues obtained from ten healthy control pregnant women and ten pregnant women with preeclampsia (accession number: GSE6573). The subjects from GSE6573 did not have a difference between age and body mass index at delivery. They presented differences in gestational age at birth, newborn weight, systolic blood pressure (BP) and diastolic BP. They did not smoke and did not have diabetes, chronic hypertension or renal disease (Table 1)^27^.

**Table 1:** GSE6573 Clinical information.

	Healthy control pregnant	Preeclamptic women
Age (years)	28 (21–37)	29 (19–38)
BMI at delivery (Kg/m^2^)	28.2 (20.3–32.4)	30.9 (24.2–35.7)
Gestational age at delivery (week)	39.0 (34.4–39.6)	33.1 (26.4–36.0)
Newborn weight (g)	3433 (2575–3695)	1735 (879–2764)
Systolic BP at delivery (mmHg)	120 (90–130)	158 (147–171)
Diastolic BP at delivery (mmHg)	78 (70–85)	105 (95–110)

Clinical and physical exam information from GEO2 libraries subjects from accession number GSE6573.

The other data came from three placentae of severely preeclamptic women with early-onset (earlier than 31 weeks gestation) and three placentas with a late-onset (31 weeks gestation or later) (accession number: GSE4707). In the original study, six subjects with early-onset type and eight subjects with late-onset type were used^28^. Unfortunately, we were unable to obtain more specific information about the patients used in the GEO dataset.

#### Established genes related to the hypertensive phenotype

The analysis was performed in DisGeNET version 6.0 (disgenet.org/) databank to assess the genes associated with the hypertension phenotype (C0020538)^29^. InteractiVenn was used to compare the shared genes between our gestational hypothyroidism DEG and hypertensive phenotype hallmarks.

From the common genes, the protein-protein interaction (PPI) network was made by STRING v.11.0 (https://string-db.org/), setting the required high confidence interaction score as 0.700. Active textmining, experiments and databases were setting as interaction sources. Reactome pathways were used in STRING to determine the most abundant pathway found^30^.

The interactive enrichment network gene analysis by ToppCluster (https://toppcluster.cchmc.org/) database was used with two gene set^31^. The first was made between common genes between gestational hypothyroidism with hypertensive hallmarks and in the second we used candidate fetal genes circulating in maternal blood suggested by Maron J. L. *et al*.^32^.

## Results

### Transcriptome library construction and genetic evaluation

We constructed nine libraries (three from healthy thyroid pregnancy, three from gestational hypothyroidism and three from nonpregnant women) and used them in this study. We obtained 78.86% to 88.66% of valid reads and the target detected ranged from 47.35% to 62.39%. No aberrant behavior was noticed in quality control analysis and the libraries also showed homogeneity.

### Circulating RNAs in healthy women, pregnancy and gestational hypothyroidism

None of the pregnant women took any drugs, smoked or consumed alcoholic beverages. All patients were negative for antithyroid antibodies at the time of blood sampling, but we did not have access to antibody information prior to pregnancy. The characteristics of pregnant and non-pregnant subjects are shown in Table 2.

**Table 2:** General epidemiological and experimental data.

	Healthy Thyroid Pregnancy (HTP)	Gestational Hypothyroidism (GHT)	Nonpregnant women (NPG)	p-value
**Clinical history**				
Age (years)	23 (22–26)	35 (22–38)	40 (36–40)	0.086
Gestational age (weeks)	38.7 (38.4–40.7)	41 (37.2–41)	—	0.507
Previous abortions	yes (66.7%)	no (100%)	—	0.114
Twinning	no (100%)	yes (33.3%)	—	0.317
Cesarean birth	1 (33.3%)	2 (66.7%)	—	0.456
Vaginal delivery	2 (66.7%)	1 (33.3%)	—	
Primigravida	1 (33.3%)	2 (66.7%)	—	0.239
Multigravida	2 (66.7%)	1 (33.3%)	—	
New born weight (g)	3515 (2695–3850)	3290 (2595–3600)	—	0.513
**Physical exam**				
Weight (Kg)	70 (66–70.6)	87.50 (63–92)	83.5 (42–138)	0.733
Systolic BP (mmHg)	110 (110–120)	118 (90–120)	110 (80–150)	0.966
Diastolic BP (mmHg)	60 (60–80)	68 (60–80)	80 (60–110)	0.582
**Laboratory tests**				
TSH (µUI/mL)	1.94 (1.69–2.86)	4.36 (3.70–4.49)	2.97 (1.34–4.23)	0.099
T4 (µUI/mL)	1 (1–0.93)	1 (0.82–1)	1.24 (1.06–1.33)	0.052

Primary information about the clinical history, physical exam and laboratory tests data from women with healthy thyroid pregnancy (HTP), gestational hypothyroidism (GHT) and nonpregnant women (NPG). The data are presented as median, minimum and maximum values or frequency and p-value.

We identified 3 DEG lists (GHT in related to HTP, HTP to NPG and GHT to NPG). In the first list, we identified 1500 DEGs reflecting in GHT alterations in related to HTP, 1226 are downregulated genes (81.73%) and 274 are upregulated genes (18.27%). In the second list, we identified 1656 DEGs reflecting in HTP alterations concerning NPG, 272 are downregulated genes (16.43%) and 1384 are upregulated genes (83.57%). In the third list, we identified 1015 DEGs reflecting in GHT alterations to NPG, 549 are downregulated genes (54.09%) and 466 are upregulated genes (45.91%).

### Trimester-specific plasma RNA fluctuation

**Figure 1:**
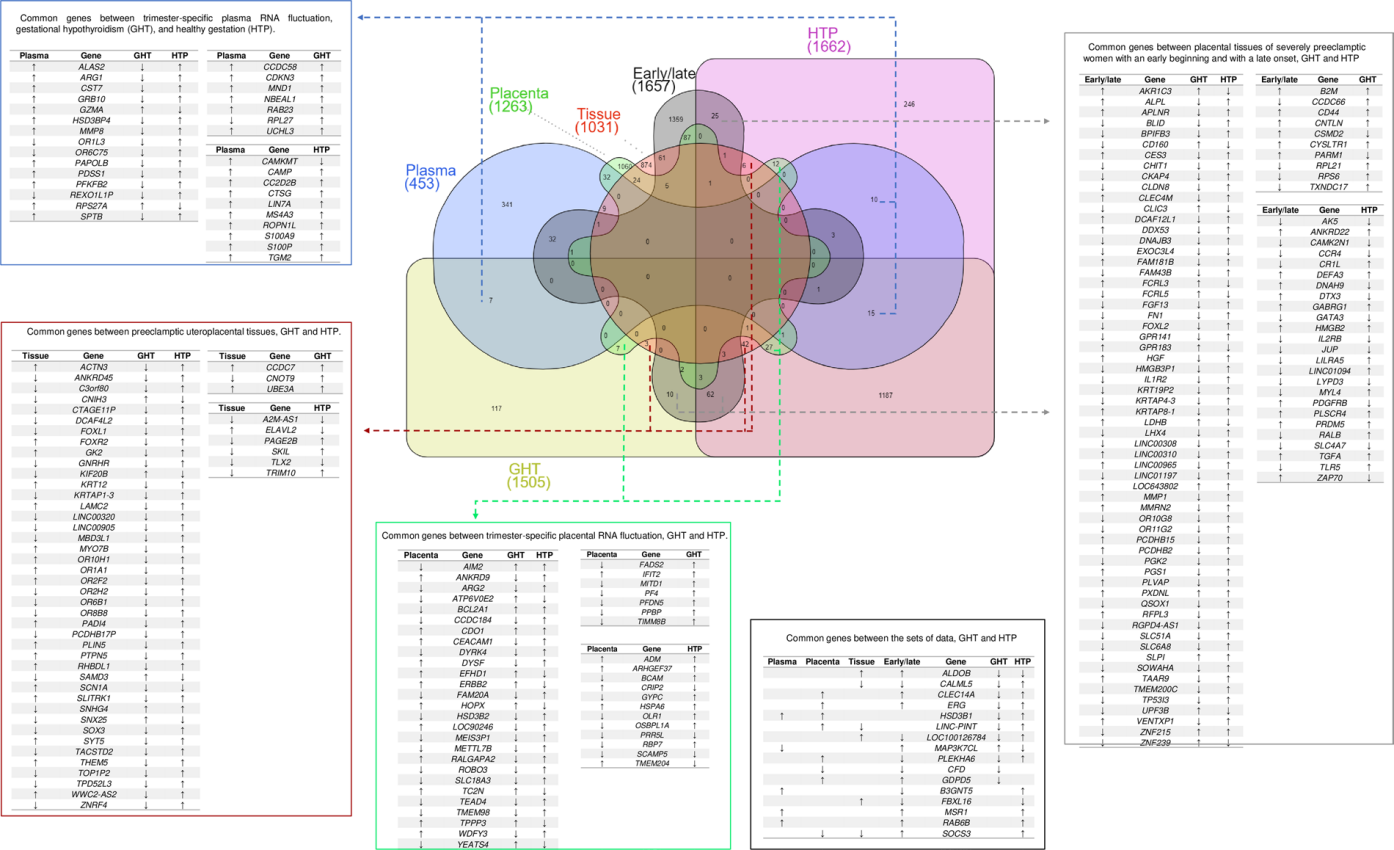
Common genes identified between the groups in the analyses. The Venn plot shows the shared genes between the groups, annotated in tables. The genes encountered in the trimester-specific plasma RNA fluctuation are represented in the horizontal Hourglass shape, trimester-specific placental RNA fluctuations are shown in the central octagon structure, placental preeclamptic tissues are in the central circular shape, and the placental tissues of severely preeclamptic women with early or late-onset are presented in the cross shape. The circulating RNA transcriptome differentially expressed genes are in two rectangular shapes. The Gestational Hypothyroidism (GHT) is in the horizontal, and the women with healthy gestation (HTP) are represented in the vertical rectangular shape.

We identified 453 DEGs in plasma RNA showing variation, but only 37 also were encountered in our circulating DEG. 15 genes were common to HTP and GHT, 13 of which were downregulated in the GHT, while 10 of these genes were upregulated in plasma RNA fluctuation in this gestational period and HTP group. In the GHT and plasma RNA fluctuation groups, we identified seven common genes and almost all of them were upregulated in these two groups. In the HTP and plasma RNA fluctuation groups, we identified ten genes and both were upregulated except one in HTP group. Five genes were shared in more than one analysis (Fig. 1).

### Trimester-specific placental RNA fluctuation

We identified 1263 DEGs in placental tissues from the second trimester and samples from term placentae from healthy women, 54 genes are common with ours. Twenty-seven placental RNA fluctuation genes were common to our circulating DEG, 17 of which were downregulated in GHT, seven in HTP and also 15 in the placental RNA fluctuation group (Fig. 1).

Regarding the shared genes between the GHT and placental RNA fluctuation groups, we identified seven genes and six of them with opposite profiles of expression. Between the HTP and placental RNA fluctuation groups, we identified twelve genes, four of which were downregulated in HTP, while seven were downregulated in the placental RNA fluctuation group. Also, eight genes were shared in more than one analysis (Fig. 1).

### Placental tissue from preeclamptic women

We identified 1031 DEGs from preeclamptic uteroplacental tissues and 1657 DEGs from placental tissues from severely preeclamptic women with an early or late-onset, in total 57 and 111 genes were common with our circulating DEG, respectively (Fig. 1).

In the preeclamptic uteroplacental tissues, 42 genes were common with GHT and HTP; in related to the expression, 37 of them in GHT, 5 in HTP and 24 in preeclamptic uteroplacental tissues were downregulated. Between only in GHT and the preeclamptic uteroplacental tissues, we identified three common genes almost all upregulated. Six were common genes between HTP and the tissues, 3 and 5 downregulated, respectively (Fig. 1).

From placental tissues from severely preeclamptic women with an early or late-onset, 62 genes were common with GHT and HTP, and we identified 36, 49 and 10 downregulated genes, respectively. Between the GHT and early/late preeclampsia groups, we identified ten common genes, 4 of which showed similar expression. Additionally, we identified 25 common genes between the HTP and early/late preeclampsia groups, 10 of which showed opposite expression patterns in the two groups (Fig. 1).

We also found 6 and 14 common genes in preeclamptic uteroplacental tissues and early/late preeclampsia onset, respectively, in more than one analysis (Fig. 1).

### Established genes related to the hypertensive phenotype

We identified 1309 genes from DisGeNET related to hypertensive disease; 62 of them were also found in GHT DEG. Some of these were associated with the hypertensive phenotype hallmarks due to changes in expression or genetic variation. 49 (79.03%) of these genes have not been described in the literature related to gestational hypothyroidism and 26 (41.93%) with gestational hypertension yet (Table 3).

**Table 3:** Shared genes between the thyroid dysfunction and hypertensive phenotype groups.

Gene	GHT	Gestational hypertension
*ADRA2B*	0	0
*ADRB1*	0	0
*APLNR*	0	0
*APOB*	2	33
*AQP1*	1	3
*ARG1*	0	1
*ARG2*	0	0
*ATP2B2*	0	0
*B2M*	0	4
*BMP2*	0	2
*BTF3P11*	0	0
*CACNA1D*	0	1
*CCL21*	0	0
*CDO1*	0	0
*CLC*	0	0
*CXCL8*	0	10
*CYP1B1*	0	0
*F2*	1	233
*F5*	1	191
*FN1*	0	7
*FOXE3*	0	0
*GAL*	2	21
*GPER1*	0	3
*GPR101*	0	0
*GSTT1*	0	10
*HAVCR1*	0	5
*HGF*	0	22
*HOPX*	0	0
*HP*	0	71
*HSD3B1*	0	3
*HSD3B2*	0	2
*IFNA2*	0	0
*IFNG*	1	10
*IGFBP3*	7	29
*IL4R*	0	5
*INS*	43	290
*ITGAM*	0	2
*JUN*	8	101
*KCNA5*	0	0
*KL*	0	7
*LAMC2*	0	0
*LTF*	0	2
*MAS1*	0	1
*MC3R*	0	0
*MMP1*	0	27
*MMP9*	1	119
*NPBWR1*	0	0
*OR10A4*	0	0
*OXTR*	0	0
*P2RX6*	0	0
*PDGFRA*	0	2
*PON1*	1	13
*PPBP*	0	1
*PSEN2*	0	0
*RETN*	1	5
*SCN10A*	0	0
*SHBG*	11	37
*SLC4A1*	0	1
*SLC6A19*	0	0
*SLPI*	0	0
*SSTR4*	0	0
*UTS2R*	0	1

Common differentially expressed genes between those associated with gestational hypothyroidism (GHT) and genes previously established to be associated with the hypertensive phenotype hallmarks, and the number of publications in the literature relating the gene to GHT or gestational hypertension.

**Figure 2:**
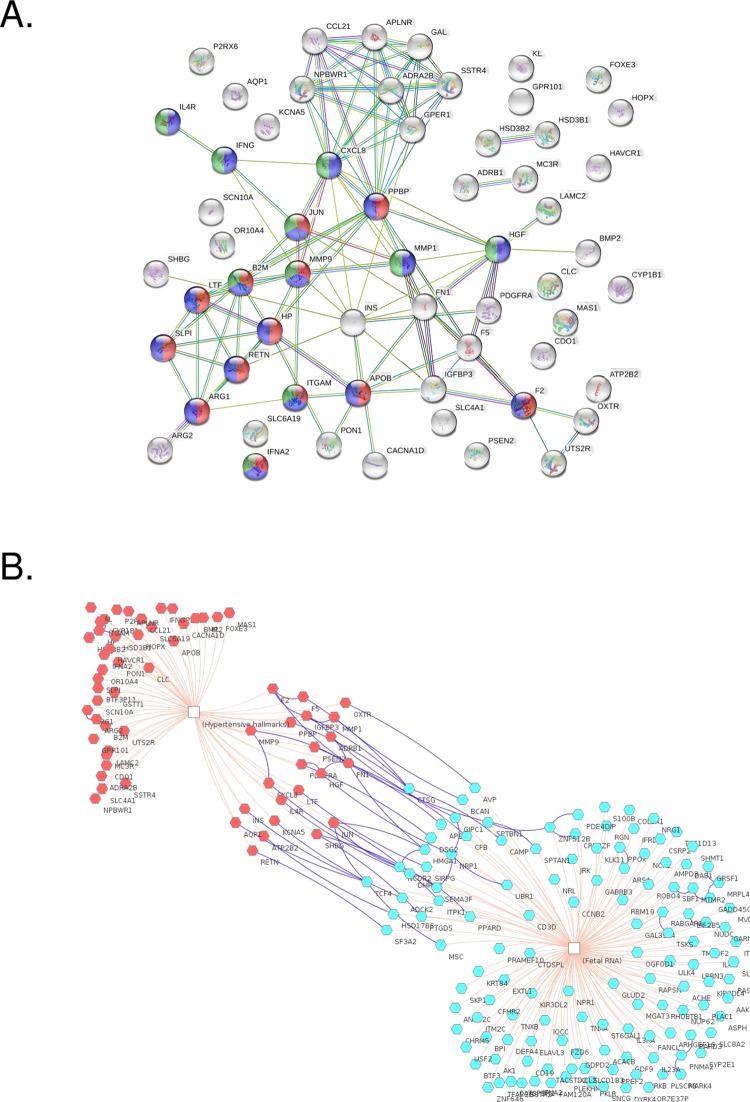
Interactive network analysis. (**A**) The protein-protein interaction (PPI) between 62 significant genes in gestational hypothyroidism with hypertensive phenotype hallmarks. The network was constructed using STRING by the required high confidence interaction score as 0.700. A total of 60 nodes were found, 115 number of edges with 3.83 average nodes degrees, 0.496 of clustering coefficient, 35 expected number of edges and a p-value <0.001. The yellow, purple, light blue and lilac lines represent the textmining, experimental data, databases and protein homology interactions, respectively. The blue nodes represent the immune system, red nodes the innate immune system and green nodes the cytokine signaling in the immune system, all with an FDR (False Discovery Rate) <0.001. (**B**) The interactive enrichment network gene was constructed by ToppCluster. The 62 common genes in gestational hypothyroidism and hypertensive phenotype hallmarks were shown in red and 131 fetal RNA is represented in light blue. 35.48% of 62 genes are related to fetal RNA.

In the protein-protein interaction (PPI) networks, 60 of 62 genes presented a total of 115 edges with 3.83 average nodes degrees, 0.496 of clustering coefficient, 35 expected number of edges and a p-value <0.001. The most predominant pathway found was related to the immune system (blue nodes), innate immune system (red nodes) and cytokine signaling in the immune system (green nodes), all with a significant FDR < 0.001 (Fig. 2A). The yellow line means the textmining score interaction source, which is derived from the co-occurrence of gene or protein names in abstracts. The purple line indicates the experimental score derived from experimental data. The light blue line means databases score derived from curated data of various databases and the lilac line represents the protein homology interaction.

In the interactive enrichment network gene analysis, we used our 62 genes in the first set and 131 out of 157 genes, representing possible fetal origin genes, as the second set. We found 22 out of 62 (35.48%) genes that interact with fetal RNAs (Fig. 2B).

## Discussion

Surprisingly, we found common genes between GHT and preeclampsia conditions. Some of these genes exhibit mechanisms related to trophoblastic invasion, vasodilatation or even preeclampsia and hypertension and these genes have the potential to serve as biomarkers in pregnant women with hypothyroidism or to be used to elucidate the mechanisms by which this disease can lead to hypertensive outcomes and preeclampsia.

One of the most plausible hypotheses for explaining preeclampsia is deficient trophoblast invasion in the placentation period. This deficiency can cause abnormalities in the uteroplacental circulation, leading to the secretion of antiangiogenic factors that induce endothelial dysfunction and triggering clinical manifestations such as hypertension. Concerning the genes involved in trophoblast invasion, we identified, for example, *S100P*, *FGF13*, *MMP1*, *MMP8* and *QSOX1*.

In a study by Tabrizi M. E. A. *et al*. and Yue X. *et al*., it was found that S100P and FGF13 may act as regulators of trophoblastic invasion during the placentation period^33,34^. Incomplete invasion of trophoblasts results, for example, in a decrease of uteroplacental blood flow, a condition usually associated with preeclampsia^33^. Barker K. J. *et al*. showed that triiodothyronine (T_3_) acting synergistically with epidermal growth factor influences the proliferation and invasion of trophoblastic cells^35^. Perhaps the thyroid hormone exhibits a function that directly affects *S100P* and *FGF13*. In our study, these genes appear in HTP DGE upregulated, while *FGF13* appears in GHT downregulated.

Genes encoding metalloproteinases (MMP) play a role in the implantation of the embryo via degradation of the extracellular matrix and promotion of angiogenesis^36^. T3 can regulate MMP2 and MMP3 expression in the extravillous trophoblast^37^. In healthy pregnancies, some MMPs, for example, MMP8 and MMP9, are upregulated in uterine epithelial cells, and preeclampsia. MMP9 is downregulated in placental tissues and MMP1 is upregulated in vasculature^38^. In our GHT analysis, *MMP1* and *MMP9* were downregulated and in HTP were upregulated in maternal blood.

Saben J. *et al*. have shown that *QSOX1* is involved in placental development and is vital for the survival of trophoblast. Additionally, QSOX1 plays a role in the formation of the extracellular matrix^39^. In this study, *QSOX1* was shown to be downregulated in GHT.

The genes involved in vasodilatation include, for example, *CEACAM1* and *APLNR*. Najjar S. M. *et al*. found in an animal model that *Ceacam1* played a role in vascular endothelial cells and the production of nitric oxide in aortae^40^. Nitric oxide can promote vasodilatation, which is very important during gestation to supply the metabolic needs of the tissues^6^. However, this gene was shown to be downregulated in GHT. Vasodilatation occurs after infusion of apelin in the blood flow, Brame A.L. *et al*. analyzed that an apelin receptor (APLNR) agonist stimulated vasodilation and inotropic actions^41^. Zhou L. *et al*. identified that *APLNR* showed a decrease in your expression in late-onset preeclampsia placental tissues^42^. This gene was downregulated in GHT; thus, it could be a trigger to the development of preeclampsia in gestational hypothyroidism.

Some genes identified in our GHT group have already been related to a preeclampsia-like phenotype, such as *GZMA*, *METTL7B* and *DYSF*^43^.

We were able to correlate 62 genes that were previously linked with hypertensive diseases with gestational hypothyroidism. These differentially expressed genes in pregnant women with hypothyroidism may favor the onset of hypertension, and ultimately, they could provide clues to the shared mechanisms of the two conditions.

We found, for example, *ARG1*, *ARG2* and *HGF*.

In a study by Shah S. F. A. *et al*., the authors suggested that a polymorphism in *ARG1* may be linked to essential hypertension^44^. ARG1 is generally expressed in macrophages, coronary endothelial cells and vascular smooth muscle aortic cells, while ARG2 is expressed in human aortic endothelial cells and human umbilical vein endothelial cells^45^. In GHT, *ARG1* and *ARG2* were downregulated.

In a study by Chaudhary P. *et al*., the invasion of HTR-8/SVneo trophoblastic cells was facilitated by HGF via MAPK/PI3K and increase MMPs levels^46^. As previously reported, it was not only some MMP*s* but also *HGF* that were found to be downregulated in GHT.

We found the immune system as the most probable pathway related to gestational hypothyroidism considering our 62 common genes between hypertensive phenotype hallmarks. The maternal immune system needs to adapt throughout pregnancy, and if this does not occur satisfactorily, it can lead to miscarriage, premature birth and preeclampsia^47^. The abnormal immune response precedes the development of preeclampsia, followed by an inflammatory reaction leading to decreased nitric oxide, vasodilation, and increased vasoconstrictors such as endothelin-1^48^. Han X. *et al*. observed that changes in the immune system may be used as a prognostic in preeclampsia^49^. Silva J. F. *et al*. demonstrated in a gestational hypothyroid animal model that the disease affects the placental trophoblastic migration and the immune profile. They also related that the presence of gestational hypothyroidism decreased *Tlr4* as well as *Mmp9* and other inflammatory markers^50^. In our GHT 52 of 62 (83.87%) common genes between hypertensive phenotype hallmarks were also downregulated. Perhaps further exploring the immune system pathway in pregnant women with hypothyroidism may also elucidate the mechanism of preeclampsia appearance.

Koh W. *et al*. analyzed that the fetal RNA concentration increases in the maternal circulation during the pregnancy, reaching 15.4% in the third trimester^25^. We found some genes that may be interacting with fetal candidate genes. Chan L. Y. *et al*. evaluated that elevated TSH levels in cord blood were associated with preeclampsia possibly due to the presence of placental insufficiency and fetal hypoxia^51^. Therefore, we can infer that hypothyroidism may be related to the development of preeclampsia due to placental insufficiency that may be reflecting in the circulating fetal RNA in the maternal bloodstream.

We consider one caveat of this study to be the small number of patients used to construct the libraries, but at the time of the study, we believed that we had to use a low number of patients to detect a large number of transcripts. We also considered the TSH levels just above the standard trimester-specific reference to be a caveat of the study, but in the initial phase of the project (drawing project point), an extensive discussion was conducted among the authors, and it was considered inappropriate to not provide pregnant women with hypothyroidism with immediate treatment. On the other hand, we were able to identify a significant number of DEGs in this population, which we consider to indicate the critical impact that thyroid function exerts. However, we also think that a substantial portion of these changes may represent an adaptation to a lower concentration of thyroid hormones.

## Conclusion

We were able to identify a relevant number of differentially expressed genes in pregnant women with hypothyroidism and a mild TSH alteration. From these DEG, we identified genes associated with the hypertensive phenotype hallmarks and compared these findings with other transcriptome libraries of plasma and placenta samples from international databanks. However, additional studies are needed to elucidate the exact mechanisms involved in gestational hypothyroidism and validate the usefulness of these circulating RNAs as biomarkers of the hypertensive complication in gestational hypothyroidism.

## Data Availability

We are grateful to all the groups that left their banks available. We also have deposited our data in the Gene Expression Omnibus (GEO) database, www.ncbi.nlm.nih.gov/geo (accession number: GSE147527).
